# Diffuse Cortical, Basal Ganglia, and Bilateral Middle Cerebellar Peduncle Diffusion Restriction in Severe Alcohol-Related Hypoglycemic Encephalopathy: A Case Report

**DOI:** 10.7759/cureus.108648

**Published:** 2026-05-11

**Authors:** Soukaina Rachidi, M'hamed Riad Amanallah, Hamza Benzakour, Yasmina Zakaria, Mohamed Chraa, Nissrine Louhab

**Affiliations:** 1 Neurology, Mohammed VI University Hospital, Faculty of Medicine and Pharmacy, Cadi Ayyad University, Marrakesh, MAR

**Keywords:** alcohol, hypoglycemia, hypoglycemic encephalopathy, magnetic resonance imaging, middle cerebellar peduncle

## Abstract

Alcohol-induced hypoglycemia is an uncommon but potentially life-threatening metabolic complication, particularly in the setting of prolonged fasting and chronic alcohol use. We report the case of a 63-year-old man with a history of alcoholism who presented in a deep coma after binge drinking and reduced oral intake. On admission, severe hypoglycemia (30 mg/dL) was documented, prompting urgent metabolic correction and neuroimaging evaluation. Brain magnetic resonance imaging (MRI) subsequently demonstrated diffuse cortical diffusion restriction with additional involvement of the neostriatum, posterior limbs of the internal capsules, and bilateral middle cerebellar peduncles, consistent with severe hypoglycemic encephalopathy. Despite prompt glucose correction and supportive management, the patient failed to recover neurologically, remained in a persistent disorder of consciousness, and ultimately died during hospitalization. This report aims to illustrate the characteristic radiological pattern and poor clinical outcome associated with alcohol-related hypoglycemic encephalopathy. This case highlights the potential for alcohol-related hypoglycemia to produce widespread cytotoxic brain injury and underscores the prognostic significance of diffuse cortical involvement on early diffusion-weighted imaging (DWI), emphasizing the importance of early recognition for prognostic assessment and clinical management.

## Introduction

Chronic and excessive alcohol intake is widely recognized as a significant risk factor for hypoglycemia. The glucose-lowering effect of ethanol is primarily mediated through inhibition of hepatic gluconeogenesis and, to a lesser extent, increased insulin secretion [1,2]. This phenomenon is especially relevant in the setting of prolonged fasting or malnutrition, when depleted hepatic glycogen stores make endogenous glucose production heavily dependent on gluconeogenesis [3]. Consequently, alcohol-related hypoglycemia may be easily overlooked in patients presenting with altered consciousness.

Hypoglycemic encephalopathy is a severe neurological syndrome defined by sustained and profound impairment of consciousness attributable solely to hypoglycemia, after exclusion of other causes, and may result in irreversible brain injury or death, particularly when hypoglycemia is profound and prolonged [4].

We report the case of a 63-year-old man with a history of chronic alcohol use who developed severe hypoglycemic encephalopathy following binge drinking and prolonged fasting, with brain magnetic resonance imaging (MRI), including diffusion-weighted imaging (DWI), fluid-attenuated inversion recovery (FLAIR), and apparent diffusion coefficient (ADC) sequences, demonstrating extensive cortical diffusion restriction and bilateral middle cerebellar peduncle involvement. This report aims to illustrate the radiological pattern and unfavorable clinical outcome of alcohol-related hypoglycemic encephalopathy. It also highlights the importance of early recognition of characteristic MRI findings, which may provide valuable prognostic information in severe cases.

## Case presentation

A 63-year-old man with a longstanding history of chronic alcohol consumption was brought to the emergency department after being found unresponsive at home. Family members reported a recent episode of heavy alcohol intake followed by approximately 24 hours of minimal oral intake. The exact duration of hypoglycemia before discovery could not be determined, although prolonged fasting and persistent unresponsiveness before admission suggested sustained metabolic injury. On initial assessment, capillary blood glucose was critically low at 0.30 g/L (30 mg/dL). Intravenous administration of 50 mL of 50% dextrose was given prior to hospital arrival; however, no improvement in mental status was observed.

On admission to the intensive care unit, the patient was deeply comatose with a Glasgow Coma Scale (GCS) score of 3/15. Oxygen saturation was 93% while receiving 4 L/min of supplemental oxygen, and the respiratory rate was 20 breaths per minute. Hemodynamic parameters were stable. Pupillary examination revealed bilateral miosis with preserved light reactivity. Neurological evaluation was limited by the depth of coma. There was no motor response to painful stimuli in any extremity, and muscle tone was diffusely decreased. Plantar responses were minimally elicitable bilaterally. Brainstem reflexes were preserved. Routine laboratory investigations were otherwise unremarkable. Serum electrolytes and inflammatory markers were within normal limits, with the exception of profound hypoglycemia (0.30 g/L; 30 mg/dL) (Table 1).

**Table 1:** Laboratory findings on admission

Parameter	Value	Unit	Reference Range
Glucose	30	mg/dL	70–100
Hemoglobin	14	g/dL	13–17
White blood cells (WBC)	5	×10⁹/L	4–10
Platelets	224	×10⁹/L	150–400
Sodium	138	mmol/L	135–145
Potassium	4	mmol/L	3.5–5.0
Creatinine	1	mg/dL	0.6–1.3
C-reactive protein (CRP)	8	mg/L	0–5
AST (aspartate aminotransferase)	20	U/L	10–40
ALT (alanine aminotransferase)	18	U/L	7–40
Ammonia	15	µmol/L	11–35

**Figure 1:**
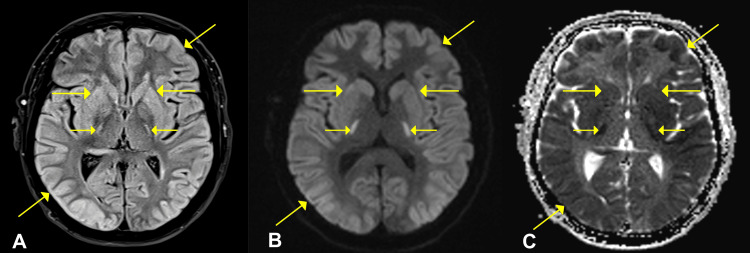
Axial brain MRI showing diffuse cortical and striatal abnormalities: (A) FLAIR demonstrating bilateral cortical hyperintensity with involvement of the neostriatum and posterior limbs of the internal capsules, (B) DWI showing symmetric diffusion restriction involving the cortex, neostriatum, and posterior limbs of the internal capsules, and (C) ADC map confirming corresponding diffusion restriction, consistent with severe hypoglycemic encephalopathy. DWI: diffusion-weighted imaging; FLAIR: fluid-attenuated inversion recovery; ADC: apparent diffusion coefficient

**Figure 2:**
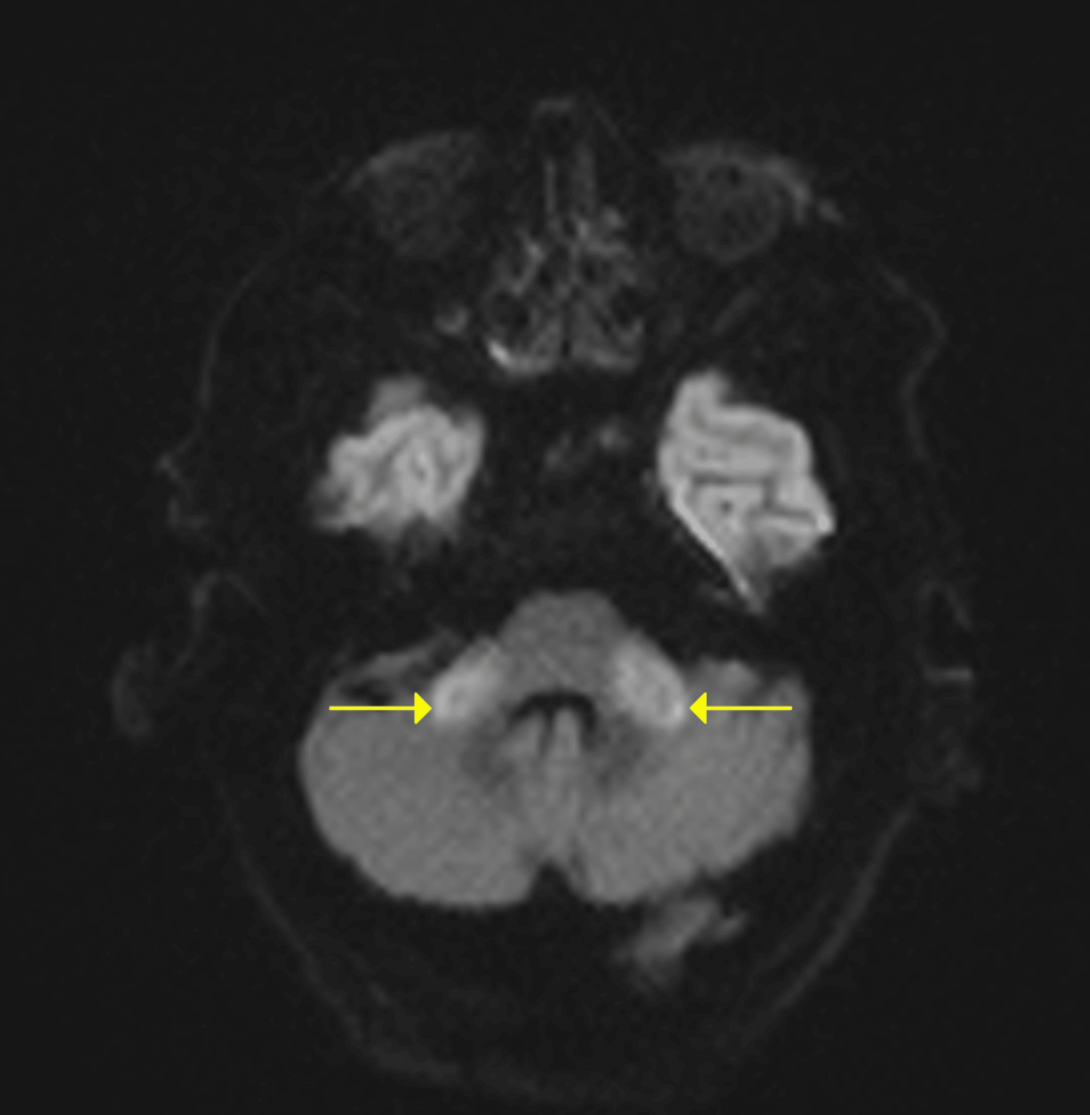
Axial DWI MRI demonstrating bilateral hyperintensity of the middle cerebellar peduncles (arrows), consistent with diffusion restriction in severe hypoglycemic encephalopathy. MRI: magnetic resonance imaging; DWI: diffusion-weighted imaging

Blood glucose normalization was achieved shortly after admission following intravenous glucose administration, while brain MRI was performed within the first 24 hours after correction of hypoglycemia due to persistent coma. MRI of the brain demonstrated extensive bilateral and symmetrical signal abnormalities, characterized by hyperintensity on DWI and FLAIR sequences, with corresponding low ADC values. The lesions involved the cortical ribbon, neostriatum (caudate nuclei and putamina), posterior limbs of the internal capsules (Figure 1), and bilateral middle cerebellar peduncles (Figure 2). This pattern of widespread cortical and subcortical diffusion restriction was consistent with severe metabolic brain injury secondary to profound hypoglycemia.

Electroencephalography (EEG) demonstrated diffuse slowing without epileptiform activity or evidence of non-convulsive status epilepticus. Hepatic encephalopathy was considered unlikely given the absence of liver failure, normal ammonia levels (15 µmol/L), and lack of imaging or clinical findings suggestive of cirrhosis. Wernicke’s encephalopathy was also excluded based on the absence of characteristic ophthalmoplegia, ataxia, or typical MRI abnormalities. The clinical and radiological findings, including the bilateral middle cerebellar peduncle lesions, were attributed to severe hypoglycemic encephalopathy in the setting of alcohol abuse and prolonged fasting. Despite correction of hypoglycemia and supportive management, including intravenous glucose administration and early thiamine supplementation, the patient failed to demonstrate neurological recovery and remained in a persistent disorder of consciousness with preserved brainstem function. The clinical course was subsequently unfavorable, and the patient died during hospitalization.

## Discussion

Alcohol-induced hypoglycemia is considered an uncommon but clinically significant complication of acute or chronic alcohol consumption, with reported rates of severe hypoglycemia below 1% among intoxicated non-diabetic individuals in emergency settings. The risk is substantially higher in vulnerable populations, including malnourished individuals, chronic binge drinkers, and patients with diabetes, liver dysfunction, or endocrine deficiencies [5,6].

Ethanol metabolism in the liver, primarily via alcohol dehydrogenase and aldehyde dehydrogenase, leads to a marked increase in the NADH/NAD⁺ ratio. This altered redox state inhibits key enzymatic steps in gluconeogenesis, particularly the conversion of lactate to pyruvate and malate to oxaloacetate, thereby impairing endogenous glucose production [7,8]. Although glycogenolysis is initially preserved, hepatic glucose output becomes critically dependent on gluconeogenesis once glycogen stores are depleted. Consequently, during prolonged fasting, when glycogen reserves are exhausted, this metabolic inhibition markedly increases the risk of severe and sustained hypoglycemia in individuals consuming alcohol. Additional contributory mechanisms may include impaired counterregulatory hormonal responses and, in some contexts, enhanced insulin-mediated effects [6,9].

Hypoglycemic encephalopathy results from critically reduced serum glucose levels, causing neuronal energy failure, intracellular ATP depletion, and impaired membrane ionic pump function. Sustained hypoglycemia leads to cytotoxic edema and restricted diffusion on DWI with corresponding ADC reduction. Although ketone bodies may transiently support cerebral metabolism, prolonged hypoglycemia ultimately triggers excitotoxic neuronal injury mechanisms resembling ischemic damage. Selective neuronal vulnerability predominantly affects metabolically active gray matter structures, including the cerebral cortex, hippocampi, and basal ganglia, while white matter tracts such as the internal capsules and middle cerebellar peduncles may also be involved in severe cases [4,10].

The earliest and most sensitive MRI abnormality in hypoglycemic encephalopathy is restricted diffusion on DWI with corresponding ADC hypointensity, often preceding changes on other sequences. Lesions are typically bilateral but may be asymmetric, and their extent correlates with the severity and duration of hypoglycemia [11]. Three principal patterns have been described: absence of visible lesions; focal involvement of highly directional white matter tracts, most commonly the internal capsule; and diffuse bilateral hemispheric white matter lesions involving the internal capsule, corona radiata, and centrum semiovale [11-13]. Cortical and basal ganglia involvement may occur, particularly in more severe or prolonged hypoglycemia, and may become more conspicuous on follow-up imaging.

Table 2 summarizes typical imaging patterns and their prognostic implications in hypoglycemic encephalopathy.

**Table 2:** Comparison of the three principal diffusion-weighted imaging (DWI) patterns in hypoglycemic brain injury Table created using Microsoft PowerPoint (Microsoft Corporation, Redmond, Washington).

Pattern	Distribution	Structures Involved	Imaging Features (DWI/ADC)	Clinical Correlation	Prognosis
Focal White Matter Pattern	Localized	Internal capsule, corona radiata, centrum semiovale	Focal diffusion restriction, often unilateral or asymmetric	Transient deficits, may mimic a lacunar stroke	Favorable, often reversible
Diffuse Hemispheric Pattern	Bilateral, widespread	Cerebral cortex + subcortical white matter	Extensive cortical and subcortical DWI hyperintensity with ADC reduction	Severe encephalopathy, coma	Poor prognosis, risk of permanent injury
Selective Gray Matter Pattern	Symmetric, deep structures	Basal ganglia, hippocampi, cerebral cortex	Symmetrical diffusion restriction in metabolically active gray matter	Altered consciousness, seizures	Variable, depends on the duration of hypoglycemia

The imaging findings observed in our patient most closely corresponded to the diffuse hemispheric pattern, which is typically associated with severe encephalopathy. MRI demonstrated diffuse cortical and neostriatal hyperintensities on DWI and FLAIR sequences with corresponding ADC reduction, consistent with extensive cytotoxic edema. Additional restricted diffusion in the bilateral internal capsules indicated concomitant involvement of major white matter tracts. Bilateral middle cerebellar peduncle diffusion restriction was also observed. Although the cerebellum is generally considered relatively spared in hypoglycemic encephalopathy due to differential glucose metabolism and transport efficiency, rare cases of middle cerebellar peduncle involvement have been reported, likely reflecting intramyelinic edema of pontocerebellar fibers in the setting of severe or recurrent hypoglycemia [14]. Although bilateral middle cerebellar peduncle involvement may also occur in other toxic-metabolic disorders, the predominance of diffuse cortical, neostriatal, and internal capsule diffusion restriction, together with the documented profound hypoglycemia and absence of imaging features suggestive of alternative etiologies such as Wernicke’s encephalopathy or hepatic encephalopathy, strongly favored hypoglycemic encephalopathy in our patient. Diffuse cortical and basal ganglia involvement on DWI has consistently been associated with unfavorable neurological outcomes, particularly when accompanied by extensive white matter tract abnormalities. In contrast to focal internal capsule lesions, which are often reversible following prompt glucose correction, widespread cortical and striatal diffusion restriction generally reflects irreversible neuronal injury. The absence of neurological recovery in our patient, who remained deeply comatose with a persistent GCS score of 3/15 despite metabolic correction, is therefore concordant with previously reported poor prognostic indicators in severe hypoglycemic encephalopathy. Persistent disorder of consciousness in this setting likely reflects extensive excitotoxic damage to vulnerable cortical networks.

Prior studies have demonstrated that early DWI patterns carry significant prognostic value in hypoglycemic encephalopathy. The absence of lesions or isolated focal internal capsule involvement is typically associated with rapid and complete recovery, whereas diffuse hemispheric white matter lesions predict poor short-term outcomes [12]. Moreover, extensive cortical and neostriatal involvement has been consistently linked to dismal prognosis, particularly when lesions fail to regress on follow-up imaging [11]. The widespread cortical, striatal, and bilateral internal capsule diffusion restriction observed in our patient aligns with these established markers of severe injury and likely accounts for the persistent disorder of consciousness despite metabolic correction.

This study has several limitations. First, as a single-case report, the findings may not be generalizable to broader patient populations. Second, long-term neurological follow-up could not be assessed due to the patient’s death, precluding assessment of long-term neurological outcome and radiological evolution. Third, the absence of serial MRI examinations limits the evaluation of lesion progression and potential reversibility over time. Although follow-up cranial computed tomography demonstrated persistent abnormalities without significant evolution, repeat MRI could not be performed during hospitalization. Despite these limitations, this report provides valuable clinical and radiological insights into severe hypoglycemic encephalopathy associated with alcohol use.

## Conclusions

Alcohol-induced hypoglycemia, particularly in the setting of prolonged fasting, can result in severe and irreversible hypoglycemic encephalopathy, as illustrated in this patient. Extensive diffusion restriction involving the cortical ribbon, neostriatum, posterior limbs of the internal capsules, and bilateral middle cerebellar peduncles on early MRI was associated with poor neurological recovery despite prompt glucose correction. Early MRI recognition of diffuse cortical and basal ganglia involvement should alert clinicians to a potentially poor prognosis and may help guide clinical decision-making and family counseling. Although early recognition and metabolic intervention remain essential, prognosis may remain unfavorable in cases with extensive cortical and deep gray matter involvement.
